# The effect of SGLT2 inhibitors on hepatic steatosis detected by MRI-PDFF in patients with type 2 Diabetes mellitus and metabolic-associated steatotic liver disease

**DOI:** 10.1007/s11739-025-03902-w

**Published:** 2025-03-14

**Authors:** Mona Ahmed Amin, Noha Adly Sadik, Hala Ahmed Saad, Mohammed Fawzy, Hend Abdallah Elsheimy

**Affiliations:** 1 Faculty of Medicine, Internal Medicine Department, Hepatology and Gastroenterology, Endocrinology and Diabetes Division, Cairo University, Cairo, Egypt; 2 Department of Diagnostic Radiology, National Hepatology and Tropical Research Institute, Cairo, Egypt

**Keywords:** MASLD, MRI-PDFF, SGLT2 inhibitors, Type 2 diabetes

## Abstract

Sodium-glucose co-transporter type-2 (SGLT2) inhibitors have been identified to have a crucial hepatoprotective role in patients with type 2 diabetes (T2DM) and metabolic-associated steatotic liver disease (MASLD). Thus, we aimed to assess the effect of SGLT2 inhibitors on hepatic steatosis in patients with T2DM and MASLD added to the standard of care (SOC) treatment. Our study was a single-arm clinical trial with trial no ISRCTN85961860. Thirty T2DM patients with MASLD were recruited from the outpatient endocrinology and diabetes clinic of the Internal Medicine Department at Kasr Al-Aini Hospital, Cairo University, Egypt. Our Patients received Empagliflozin 10 mg daily which was added to SOC treatment and followed up for 24 weeks. Magnetic resonance imaging proton density fat fraction (MRI-PDFF) was done at baseline and after 24 weeks to assess the percentage change in hepatic fat mass. Also changes in Fib-4 and NAFLD fibrosis scores were calculated. Our study showed a statistically significant decrease in the mean MRI-PDFF measurement of hepatic steatosis after 24 weeks of adding empagliflozin to SOC treatment (13.297 ± 7.15) compared to the mean at baseline (15.288 ± 8.72), *P* = 0.006 with overall percentage decrease about 13.16% of liver steatosis. There were significant decreases in BMI, fasting blood glucose, and Alanine transaminase, (*P* < 0.001, 0.03, 0.01) respectively. There were no significant differences in Fib-4 or NAFLD fibrosis scores. Adding empagliflozin 10 mg to the standard treatment in patients with diabetes and MASLD could reduce hepatic fat mass significantly after 24 weeks of treatment. Thus, adding SGLT2 inhibitors to the clinical practice guidelines could be a therapeutic agent for patients with MASLD and T2DM.

## Introduction

Metabolic dysfunction-associated steatotic liver disease (MASLD), formerly known as non-alcoholic fatty liver disease (NAFLD), is a new term to describe steatotic liver disease (SLD) associated with one or more cardiometabolic risk factors and in the absence of harmful alcohol intake. The spectrum of MASLD includes steatosis, metabolic dysfunction-associated steatohepatitis (MASH, formerly known as NASH), fibrosis, cirrhosis, and MASH-related hepatocellular carcinoma [1]. Epidemiological studies have reported a worldwide prevalence of MASLD of more than 50% in type 2 diabetic patients [2]. MASLD represents a complication and a risk factor for developing T2DM [3] which is accompanied by various complications, such as cardiovascular and chronic kidney diseases thereby seriously affecting the life expectancy in patients with T2DM [4, 5]. Therefore, early intervention and targeted treatment of MASLD in patients with T2DM are required. Several antidiabetic therapies have been investigated in the treatment of MASLD with varying results including metformin [6], glucagon-like peptide‐1 receptor agonists (GLP- 1RAs), and Sodium-glucose co-transporter type 2 (SGLT2) inhibitors [7].

SGLT2 inhibitors are novel oral hypoglycemic drugs that have received attention due to their unique mechanism of inhibiting glucose reabsorption in the proximal renal tubules and increasing urinary glucose excretion. SGLT2 inhibitors are not dependent on insulin and are associated with decreased body weight [8]. SGLT2 inhibitors have been FDA-approved as beneficial drugs in diabetic patients with cardiovascular and renal disease due to their cardiovascular and renal protective effects [9, 10]. Recently, clinical evidence has suggested that SGLT2 inhibitors have hepatoprotective effects, reducing the liver fat mass and improving the liver enzymes in patients with type 2 diabetes and MASLD [11–13]. The decrease in low-grade inflammation and oxidative stress associated with SGLT2 inhibitor therapy have been suggested as possible pathophysiological causes of improvement of hepatic steatosis [14–16]. The American Association of Clinical Endocrinology and the Japanese Society of Gastroenterology recommended adding SGLT2 inhibitors as an auxiliary therapy in the clinical practice guidelines for treating patients with type 2 DM and MASLD [17, 18]. Although SGLT2 inhibitors are considered beneficial and safe for patients with liver diseases, it is still not FDA-approved for the treatment of MASLD. Thus, our study aimed to assess the effect of SGLT2 inhibitor on liver steatosis measured by Magnetic resonance imaging Proton Density Fat Fraction (MRI-PDFF) in patients with type 2 diabetes and MASLD.

## Material and methods

### Study design

Our study was a single-arm clinical trial that included 30 adult T2DM patients with MASLD. We enrolled our patients according to sample size calculation for a clinical trial; with 0.05 alpha error and power of the study 0.80, to calculate the minimal sample size needed to detect the effect of SGLT2 inhibitors on hepatic steatosis detected by MRI-PDFF relying on the effect size found in the study by *Mohammad *et al. [19].

### Patients

**Fig. 1:**
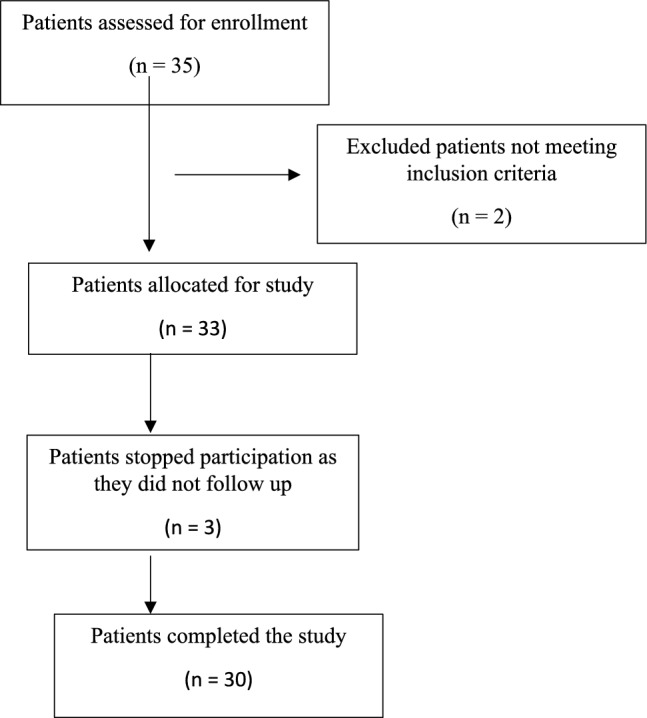
Flow chart for our single-arm clinical study

Our patients were recruited from the outpatient Endocrinology and Diabetes clinic of the Internal Medicine department, Kasr Alainy Hospitals, Cairo University, Egypt, from May 2022 to October 2023. We included adult patients with type 2 diabetes mellitus with a disease duration of 4 to 5 years with sonographic evidence of hepatic steatosis with or without elevated liver enzymes diagnosed as MASLD. Patients were already on oral hypoglycemic drugs such as metformin 1000 mg daily, Sulphonyl urea, and statin therapy before enrollment. The doses were not changed throughout the study to avoid interference with our results. Our patients were not on diet control or exercise programs. *Exclusion criteria:* We excluded T2DM patients already on SGLT2 inhibitors, Type 1 diabetes, patients with estimated GFR < 30 ml/min, pregnant and lactating females, patients with liver cirrhosis, or patients who could not tolerate SGLT2 inhibitors. Our patients received an SGLT2 inhibitor in the form of empagliflozin 10 mg daily (as the minimum effective dose approved for T2DM treatment) added to their standard of care treatment and followed up for 24 weeks with a full assessment of hepatic steatosis and fibrosis done by MRI-PDFF at the beginning of the study and after 24 weeks of adding empagliflozin. Patients were requested to visit the outpatient clinic monthly to assess any side effects and adherence. We did not report any side effects with the use of Empagliflozin 10 mg in our study. Figure 1 shows a flow chart for the study.

### Ethical aspects

The study agreed with the Helsinki Ethical Declaration 1975 guidelines. Written informed consent was obtained from the patients after ethical committee approval by the Internal Medicine Research Ethical Committee department, Cairo University with ethical approval code: (MS-663-2021).

### Methods

All subjects were subjected to thorough history taking, and clinical examination, weight, height, and waist circumference were measured. Body mass index (BMI) was calculated. Laboratory investigations in the form of serum fasting blood glucose (FBG), 2 h postprandial blood glucose (2 h pp glucose), glycated hemoglobin (HbA1C), serum alanine transaminase (ALT), aspartate transaminase (AST), creatinine, total cholesterol (TC), low-density lipoprotein (LDL-C), high-density lipoprotein (HDL-C), and triglycerides (TG) were measured. Estimated glomerular filtration rate (eGFR), Fib-4, and NAFLD fibrosis scores were calculated. Abdominal ultrasound and MRI-PDFF were performed in all patients. All the laboratory and imaging studies were performed at the baseline and 24 weeks after adding SGLT2 inhibitors.

### Abdominal ultrasound

Was done by a trained radiologist using a high-resolution B-mode ultrasonography General Electric (GE) machine 3.5 MHz probe. Hepatic steatosis was diagnosed according to the following features: (a) increased liver brightness in contrast to the kidney, (b) impaired or no visualization of the portal vein wall, and (c) impaired appearance of the diaphragm. Staging of fibrosis degree was done according to Fib-4 and NAFLD fibrosis score (NFS) [20, 21].

$$FIB - 4 = {\text{Age }}\left( {\left[ {{\text{yr}}} \right] \, \times {\text{ AST }}\left[ {{\text{U}}/{\text{L}}} \right]} \right) \, / \, \left( {\left( {{\text{PLT }}\left[ {{1}0\left( {9} \right)/{\text{L}}} \right]} \right) \, \times \left( {{\text{ALT }}\left[ {{\text{U}}/{\text{L}}} \right]} \right)\left( {{1}/{2}} \right)} \right).$$.$${\text{NAFLD fibrosis score }} = \, - {1}.{675 } + \, 0.0{37 } \times {\text{ age }}\left( {{\text{year}}} \right) \, + \, 0.0{94 } \times {\text{ BMI }}\left( {{\text{kg}}/{\text{m}}^{{2}} } \right) \, + { 1}.{13 } \times {\text{ IFG}}/{\text{diabetes }}\left( {{\text{yes }} = { 1},{\text{ no }} = \, 0} \right) \, + \, 0.{99 } \times {\text{ AST}}/{\text{ALT ratio }} - \, 0.0{13 } \times {\text{ platelet count }}\left( { \times {1}0^{{9}} /{\text{L}}} \right) \, - \, 0.{66 } \times {\text{ albumin }}\left( {{\text{g}}/{\text{dL}}} \right).$$.

### MRI protocol

Magnetic resonance imaging (MRI) is an advanced imaging technique that uses a wide range of contrast mechanisms to facilitate the detection and quantification of liver fat content. This is achieved through direct measurement of proton signal in water and fat. Traditional qualitative methods, including in-phase and opposed-phase imaging or fat-suppression techniques such as T1-weighted gradient-echo and T2-weighted fast spin-echo sequences, have previously been used. However, these methods have proven to be inadequate for quantitative assessment due to the presence of multiple confounding factors. To determine the level of liver fat content, it is essential to separate and measure the proton signals of water and fat. By doing so, a normalized fat signal ratio can be calculated, referred to as Proton Density Fat Fraction (PDFF). The PDFF is calculated by the ratio of un-confounded signals from protons within mobile triglycerides (F) to mobile water molecules (W), i.e., PDFF = F/(W + F) which is determined as a percentage. The MRI-PDFF showed a strong correlation with liver fat content that has been established by histopathologic examination, with a correlation coefficient of *r* = 0.743 (*P* < 0.001) [22].

### Statistical analysis

Data were coded and entered using the statistical package for the Social Sciences Stata version 14.2. Data was summarized using mean and standard deviation for quantitative variables and frequencies (number of cases) and relative frequencies (percentages) for categorical variables. Comparisons between pre and post-treatment were done using paired *t*-tests. Correlations between quantitative variables were done using the Pearson correlation coefficient. P-values less than 0.05 were considered statistically significant.

## Results

Our study included 30 T2DM patients with MASLD, 23 were females (76.7%) and 7 were males (23.3%). Their age ranged from (31- 63 years), with a mean age of 49.6 years. Among the included patients, 53% of them were hypertensive and five patients reported to have coronary vascular disease. Table 1 shows the anthropometric parameters, laboratory data, and the calculated Fib-4, and NAFLD fibrosis scores before and after adding Empagliflozin. There was a statistically significant decrease in the mean body weight after treatment (86.7 ± 14.99 kg) compared to the mean before treatment (90.367 ± 14.33 kg), *P* < 0.001. Also, there was a statistically significant decrease in the mean BMI after treatment (33.313 ± 5.413 kg/m^2^) compared to the mean before treatment (35.143 ± 5.55 kg/m^2^), *P* < 0.001. There was no statistically significant decrease in the mean waist circumference before and after adding empagliflozin (*P* = 0.295). Also, there were no statistically significant differences in mean systolic and diastolic blood pressure before and after treatment, *P* > 0.05. Regarding the liver enzymes, the mean ALT decreased significantly after treatment (22.96 ± 13.48 IU/L) compared to the mean before (28.8 ± 13.604 IU/L), *P* = 0.03, while the mean AST was not affected significantly, *P* = 0.07. Regarding the blood glucose indices, there was a statistically significant decrease in the mean fasting blood glucose levels after treatment (153.72 ± 44.53 mg/dl) compared to the mean before treatment (180.334 ± 65.34 mg/dl), *P* = 0.017. However, there was no significant decrease in 2 h PPG or HbA1C after 6 months of treatment (*P* = 0.452, 0.3954) respectively. Also, there was no significant difference in the lipid profile indices after treatment, (*P* > 0.05) (Table 1). Regarding the change in the stages of fibrosis scoring system and NFS**,** our study showed non-significant decrease in the mean Fib4 score after treatment (0.935 ± 0.508) compared to mean before treatment (0.955 ± 0.464), (*P* = 0.852) and in the mean NFS score after (0.397 ± 1.898) compared to mean before (0.408 ± 1.921) treatment, (*P* = 0.966) (Table 1).

**Table 1:** Comparison of anthropometric and laboratory measurements, and calculated Fib-4, and NAFLD fibrosis scores in T2DM patients with MASLD at baseline and after 24 weeks of adding SGLT2 inhibitor Empagliflozin

Variable	Mean at baseline ± SD	Mean after 24 weeks of adding SGLT2 inhibitor ± SD	*P* value
Weight (Kg)	90.367 ± 14.33	86.7 ± 14.99	< 0.001*
BMI (Kg/m^2^)	35.143 ± 5.55	33.313 ± 5.413	< 0.001*
Waist circumference (cm)	111.034 ± 12.00	108.534 ± 17.98	0.295
SBP (mm Hg)	124.500 ± 15.10	122 ± 10.71	0.35
DBP (mm Hg)	76.500 ± 9.57	77.666 ± 7.73	0.546
ALT (IU/L)	28.8 ± 13.604	22.96 ± 13.48	0.03*
AST (IU/L)	28.03 ± 11.981	22.6 ± 12.31	0.07
FBG (mg/dl)	180.334 ± 65.34	153.72 ± 44.53	0.017*
2 h PP glucose (mg/dl)	220.56 ± 63.81	209.267 ± 66.94	0.452
HbA1c %	7.787 ± 1.96	7.678 ± 1.895	0.3954
Triglyceride (mg/dl)	199.13 ± 119.41	178.41 ± 99.10	0.18
Total cholesterol (mg/dl)	203.72 ± 50.763	204.036 ± 48.761	0.96
LDL-C (mg/dl)	118.63 ± 47.61	123.93 ± 46.93	0.64
Non- HDL-C (mg/dl)	152.31 ± 53.25	154.93 ± 49.54	0.78
HDL-C (mg/dl)	45.5 ± 13.203	44.448 ± 10.06	0.791
NFS score	0.408 ± 1.921	0.397 ± 1.898	0.966
FIB-4 score	0.955 ± 0.464	0.935 ± 0.508	0.852

*SD* standard deviation, *BMI* Body mass index, *SBP* Systolic blood pressure, *DBP* Diastolic blood pressure, *ALT* Alanine transaminase, *AST* Aspartate aminotransferase, *FBG* Fasting blood glucose, *2 h PP* 2 h postprandial blood glucose, HbA1c (glycated hemoglobin), *LDL-C* low density lipoprotein cholesterol, *HDL-C* high density lipoprotein cholesterol, *Non- HDL-C* non- high density lipoprotein cholesterol, *NFS* NAFLD fibrosis score, *FIB-4 Score* Fibrosis 4 score

**Fig. 2:**
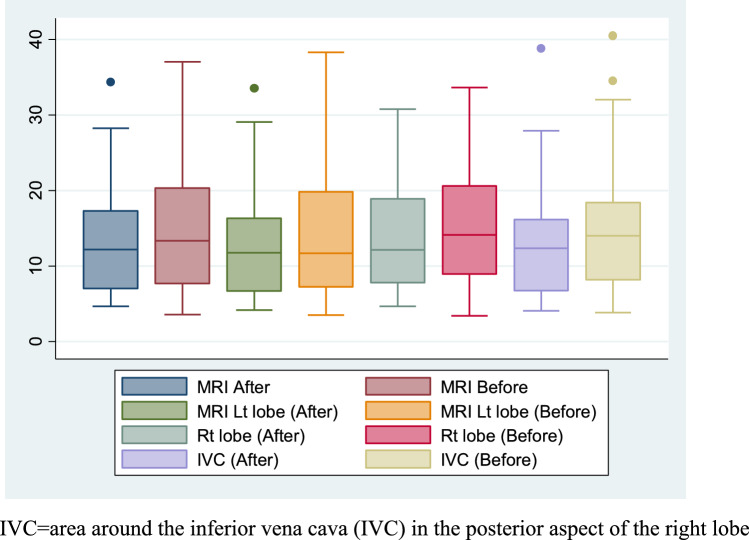
Box Plot of MRI-PDFF Readings before and after adding SGLT-2 inhibitor

By comparing the mean MRI-PDFF readings before and after adding Empagliflozin (which is the calculated mean of the right lobe, left lobe, and the posterior aspect of the right lobe), we found a significant decrease in the fat content by 13.16% after adding Empagliflozin with mean (13.28 ± 7.15) compared to the mean at baseline (15.289 ± 8.72), *P* = 0.006. The most significant reduction was in the posterior aspect of the right lobe around the inferior vena cava (IVC), *P* = 0.005 (Table 2). This indicates that the treatment likely had a statistically significant effect on the MRI measurements with an overall percentage decrease of about 13.16% of liver steatosis 24 weeks after adding Empagliflozin to standard of care treatment. Figure 2 demonstrates the change in the percentages of liver steatosis in diabetic patients measured by MRI_PDFF in the right lobe, left lobe, and area around IVC in the posterior aspect of the right lobe at baseline and after treatment with SGLT2 inhibitor Empagliflozin 10 mg for 24 weeks.

**Table 2:** Comparison of mean MRI-PDFF readings at baseline and after 24 weeks of adding SGLT-2 inhibitor in T2DM patients with MASLD

Variable	Mean before *SD	Mean after SD	Percentage of change	*p* value
Mean MRI	15.289 ± 8.72	13.28 ± 7.15	– 13.16%	0.006*
MRI Right Lobe	15.76 ± 8.53	13.921 ± 7.05	– 11.65%	0.021*
MRI Left Lobe	14.379 ± 9.09	12.713 ± 7.01	– 10.44%	0.056
MRI of posterior aspect of right lobe	15.728 ± 8.93	13.260 ± 7.67	– 15.66%	0.005*

P* is significant, *MRI-PDFF* Magnetic resonance imaging-proton density fat fraction, *SD Standard deviation, mean MRI, is the calculated mean of the right lobe, left lobe and the posterior aspect of right lobe

By applying the Pearson correlation, we did not report any significant correlation between the mean change of hepatic fat mass percentage at baseline and after 24 weeks of treatment and the mean change in the studied parameters among all patients (Table 3).

**Table 3:** Correlation of mean hepatic fat mass % change and mean change of the studied parameters

Mean change in studied parameters	Mean hepatic fat mass % change
Weight (Kg)	*r *= 0.148 *P* = 0.436
BMI (Kg/m^2^)	*r * = 0.067 *P* = 0.726
Waist circumference (cm)	*r *= 0.112 *P* = 0.557
SBP (mm Hg)	*r *= 0.112 *P* = 0.275
DBP (mm Hg)	*r * = 0.277 *P* = 0.139
FBG (mg/dl)	*r *= 0.013 *P* = 0.947
2 h pp glucose (mg/dl)	*r * = 0.036 *P* = 0.849
HbA1C %	*r *= – 0.036 *P* = 0.851
AST (IU/L)	*r * = 0.082 *P* = 0.666
ALT (IU/L)	*r *= 0.211 *P* = 0.263
Serum creatinine (mg/dl)	*r * = 0.128 *P* = 0.501
Triglycerides (mg/dl)	*r *= – 0.039 *P* = 0.838
Total cholesterol (mg/dl)	*r * = 0.035 *P* = 0.852
LDL-C (mg/dl)	*r * = 0.145 *P* = 0.445
HDL-C (mg/dl)	*r *= 0.152 *P* = 0.424
Non- HDL-C (mg/dl)	*r * = 0.126 *P* = 0.507
FIB-4	*r * = – 0.023 *P* = 0.904
NFS	*r * = – 0.095 *P* = 0.618

*r* Pearson correlation, *BMI* Body mass index, *SBP* Systolic blood pressure, *DBP* Diastolic blood pressure, *ALT* Alanine transaminase, *AST* Aspartate aminotransferase, *FBG* Fasting blood glucose, *2 h PP* 2 h postprandial blood glucose, HbA1c (glycated hemoglobin), *LDL-C* low density lipoprotein cholesterol, *HDL-C* high density lipoprotein cholesterol, *Non- HDL-C* non- high density lipoprotein cholesterol, *FIB-4 Score* Fibrosis 4 score, *NFS* NAFLD fibrosis score

## Discussion

The crucial role of sustained weight loss by diet control and exercise is one of the effective methods for improving fatty liver disease [23], a decrease in BMI of 5% can improve fatty liver by 25% [24]. We always discuss healthy lifestyles with our patients as the first line for prevention and management of fatty liver disease, unfortunately, most patients usually fail to adhere to diet control or to perform exercise regularly and always ask for drug treatment. Recently, clinical studies have highlighted the benefit of adding SGLT-2 inhibitors to the SOC treatment in patients with T2DM and MASLD [25]. Therefore, we aimed to assess its beneficial effect on hepatic fat mass in patients with type 2 diabetes and MASLD.

Our study found a statistically significant decrease in the mean hepatic steatosis associated with a significant reduction in BMI, fasting blood glucose, and serum ALT levels after 24 weeks of adding empagliflozin 10 mg daily. In agreement with our study, several studies showed that adding SGLT-2 inhibitors to diabetic patients improved hepatic steatosis, serum liver enzymes, body weight, and fasting blood sugar [19, 26–31]. Similarly, the meta-analysis by *Pradhan *et al. which included 9 randomized trials [32], and the meta-analysis of randomized controlled trials by *Bica *et al. which included 21 articles [33], revealed that the use of SGLT-2 inhibitors resulted in improvement of liver fat mass measured by proton density fat fraction, serum transaminases, and body weight. The E-LIFT trial included 50 T2DM patients randomly assigned to either the empagliflozin group (10 mg daily) or the standard treatment without empagliflozin for 20 weeks. The patients in the empagliflozin group experienced significant reductions in liver fat by MRI-PDFF, despite similar glycemic control [34]. Also, The EMPACEF trial studied the effects of empagliflozin 10 mg for 12 weeks on ectopic fat stores in type 2 diabetes, the researchers found that the empagliflozin 10 mg decreased the liver fat content measured by MRI [35]. Similarly, the Iranian study by *Chehrehgosha *et al. [36] showed that the use of empagliflozin 10 mg was associated with a decrease in the hepatic fat mass and fibrosis compared to the pioglitazone group after 20 weeks of treatment in the T2DM patients.

Researchers attributed the cause of the decrease in the fat content after adding SGLT-2 inhibitors to the change in the glucose and insulin levels associated with the improvement in insulin resistance [19, 37, 38]. Of note, the amelioration in hepatic fat was also detected in patients treated with other types of anti-diabetic drugs, such as metformin [6], thiazolidinediones [39], and GLP-1 Ras [7], which linked the mechanism of improvement to glycemic control*.* However, in our study, there were no significant changes in HbA1C or the 2hrsPPG despite the decrease in the hepatic steatosis and only fasting blood glucose was significantly decreased. In contrast, *Bellanti *et al. [40] reported a significant reduction after six months of treatment, a lowering effect on fasting serum glucose and HbA1c. Also, *Arase *et al. [28] revealed that the 24-week SGLT2 inhibitors treatment significantly improved glucose metabolism-related variables including HbA1c, fasting plasma glucose, fasting insulin, and HOMA. The study by *Kahl *et al. [41] that enrolled patients with T2DM and good glycemic control showed significant improvement in liver fat mass in the patient group treated with empagliflozin (25 mg) compared to patients on conventional treatment of pioglitazone and metformin or placebo group. These results strongly suggest that good glycemic control and weight loss are not the only mechanisms associated with the beneficial effects of SGLT-2 inhibitors treatment on hepatic steatosis.

Various mechanisms have been hypothesized regarding the possible pathophysiological causes of hepatic steatosis improvement with SGLT-2 inhibitors treatment. The improvement occurs in insulin resistance due to good glycemic control, the decrease in insulin levels, and the associated reduction in body weight leading to the decline in hepatic de novo lipogenesis [14]. Also, SGLT-2 inhibitors stimulate the SGLT2 receptors in alpha pancreatic cells and lead to glucagon release. Subsequently, the released plasma glucagon levels induce β-oxidation, which stimulates fatty acid oxidation instead of carbohydrate oxidation reducing the liver triglyceride content and producing improvement of hepatic steatosis [14]. Also, administration of SGLT2 inhibitors decreases collagen deposition and the inflammatory cytokines in the liver [15]. In addition to their ability to reduce glucotoxicity, SGLT-2 inhibitors reduce free radical generation, inhibit pro-oxidants, and stimulate antioxidant systems, such as superoxide dismutases (SODs) and glutathione (GSH) peroxidases [16].

Liver fibrosis has been identified as one of the most crucial contributing factors to liver-related death in diabetic patients with MASLD [42]. Thus, we analyzed the effect of SGLT2 inhibitor treatment on liver fibrosis scores in T2DM with MASLD. There was a decrease in Fib4 score and NFS score after treatment, however, the change was not statistically significant in agreement with other studies [28, 29, 31]. In contrast, *Akuta *et al. [43] reported histological improvement in the scores of fibrosis stage by 33% after treatment with canagliflozin for 24 weeks in patients with T2DM and NAFLD. Also, *Arai *et al. showed sustained improvement in the FIB-4 index after 3 years of follow-up by adding SGLT2 inhibitors [44]. Also, *Bellanti *et al. [40] investigated the effects of SGLT2 inhibitors on markers of oxidative stress, inflammation, liver steatosis, and fibrosis in 52 patients of T2D with NAFLD. The proportion of patients affected by a fibrotic form of liver disease according to NAFLD fibrosis score, and FIB-4, reduced significantly after 6 months of therapy in the SGLT2 inhibitors group, the main difference with the current study is the fact that they enrolled only patients with fibrosis. In our research, the non-significant improvement in liver fibrosis scores might be related to differences in the study design, selection of patients, dose of empagliflozin, and short follow-up duration. SGLT-2 inhibitors can play a crucial hepatoprotective role by reducing hepatic inflammation, apoptosis, and endoplasmic reticulum stress. Also, SGLT-2 inhibitors lead to decreased activation of hepatic satellite cells and the p53/p21 pathway resulting in amelioration of hepatic fibrosis and HCC development [45].

Regarding the lipid profile measurements, we did not report a significant difference in the lipid profile after adding Empagliflozin 10 mg for 24 weeks in agreement with *Seko *et al. study that used either Canagliflozin 100 mg or Ipragliflozin 50 mg for 24 weeks [30]. In contrast, *Arase *et al. [28] reported decreased serum levels of total cholesterol and triglycerides after adding SGLT2 inhibitor therapy. *Hayashi *et al. [46] showed a significant decrease in the small dense atherogenic LDL particles and an increase in the less atherogenic LDL particles after treatment with Dapagliflozin for 12 weeks which could explain the beneficial effects on cardiovascular outcomes. SGLT2 inhibitors can affect lipid metabolism in several ways that may counterbalance the associated increase in LDL levels. SGLT2 inhibitors decrease the lipid accumulation in visceral fat, reduce lipid oxidation, and shift substrate utilization towards ketone bodies utilization, which are more efficient in myocardial metabolism. Less reactive oxygen species are created through their oxidation, affecting the β-oxidation and the transportation of lipid molecules in the cells [47].

Regarding BP measurements, our study showed nonsignificant changes in blood pressure readings before and after adding empagliflozin 10 mg. There were contradictory results regarding the effect of SGLT2 inhibition on blood pressure measurements. SGLT2 inhibitors may potentially assist BP goal achievement in people with type 2 diabetes and hypertension within 3–5 mm Hg which varies slightly by specific SGLT2 inhibitor but does not reach the desired levels achieved by primary antihypertensive agents [48]. A meta-analysis of 16 RCT studies showed a significant decrease in SBP by 1.68 mmHg with no change in DBP [49]. Also, *Teo *et al. study [50] showed a mild significant reduction in systolic and a nonsignificant reduction in diastolic blood pressure. Various mechanisms were postulated for the effect of SGLT2 inhibitors on blood pressure mostly related to glucosuria, natriuresis, and weight loss [48]. The impact of SGLT2 inhibitors on blood pressure measurements is mostly based on 24-h BP monitoring, with several instructions for patients before measurements, such as salt and caffeine-containing beverages restriction during the study which was not in our study design protocol.

*Eventually, our* study had limitations as a relatively small sample size of a short follow-up duration. Also, we did not assess the effect of SGLT2 inhibitors on insulin resistance, which could reinforce our conclusions. Further controlled studies with larger sample sizes, increasing the maximum tolerated dose of empagliflozin, and longer follow-up durations are needed to confirm our results and to identify the best treatment modality. *In conclusion*, adding Empagliflozin 10 mg/day for 24 weeks in patients with T2DM and MASLD significantly reduced hepatic steatosis. Thus, SGLT2 inhibitors could be a beneficial drug therapy to be added to the clinical practice guidelines for patients with T2DM and MASLD.

## Data Availability

Data is available on request from the corresponding author.
